# Application of telemedicine system for older adults postoperative patients in community: a feasibility study

**DOI:** 10.3389/fpubh.2024.1291916

**Published:** 2024-02-16

**Authors:** Quan-Peng Wang, Wan-Ying Chang, Man-Man Han, Ye-Xiao Hu, Sai-Sai Lin, Ye-Chun Gu

**Affiliations:** ^1^General Surgery Department, Wenzhou Hospital of Integrated Traditional Chinese and Western Medicine, Wenzhou, China; ^2^Clinical Medical College, Zhejiang Chinese Medical University, Hangzhou, China

**Keywords:** aging society, postoperative, telemedicine system, telemedicine, older adults patients

## Abstract

**Purpose:**

In response to the growing challenges posed by an aging society, a telemedicine system was developed specifically for older adults postoperative patients, and its effectiveness was thoroughly investigated.

**Methods:**

Between May 2020 and May 2022, a total of 88 older adults postoperative patients were enrolled and randomly allocated into an experimental group and a control group. The experimental group received telemedicine services after discharge, while the control group received conventional medical services following the traditional protocol. One month after discharge, various indicators were evaluated for both groups, including number of visits, medical expenditures, postoperative recovery, anxiety, depression and satisfaction.

**Results:**

The number of visits and medical expenditures of the experimental group were less than those of the control group [1 (0, 1) vs. 1 (1, 2), *Z* = −3.977, *p* < 0.001; 25.25 (0.00, 277.40) yuan vs. 174.65 (49.63, 446.10) yuan, *Z* = −2.150, *p* = 0.032]. In both groups, there were 2 cases of incision infection, respectively. No significant difference was observed between the two groups (Fisher χ^2^, *p* = 0.259). In both groups, there was no instance of incision bleeding, incision dehiscence, readmission, or reoperation. Additionally, there was no significant difference in physical status between the two groups at discharge and after discharge (66.06 ± 8.92 vs. 65.45 ± 7.39 t = 0.287, *p* = 0.775; 73.33 ± 9.97 vs. 70.91 ± 7.50, *t* = 1.202, *p* = 0.235). And there was no significant difference in the change of physical status between the two groups after discharge [10.00 (0.00, 10.00) vs. 5.00 (0.00, 10.00), *Z* = −1.077, *p* = 0.281]. There was no significant difference in body weight change between the two groups after discharge [1.05 (0.38, 1.60) Kg vs. 0.80 (0.50, 1.43) Kg, *Z* = −0.265, *p* = 0.791]. There was no significant difference in the levels of anxiety and depression between the two groups at discharge (45.64 ± 8.10 vs. 44.60 ± 8.24, *t* = 0.520, *p* = 0.604, 48.33 ± 8.46 vs. 47.50 ± 6.85, *t* = 0.418, *p* = 0.677). But the levels of anxiety and depression in the experimental group were lower than those in the control group after discharge (34.92 ± 7.38 vs. 39.03 ± 8.42, *t* = −2.183, *p* = 0.032, 37.86 ± 7.29 vs. 41.93 ± 7.13, *t* = −2.281, *p* = 0.025); The change of anxiety level and depression level of the experimental group were more than those of the control group [−10.00 (−11.25, −8.75) vs. −5.00 (−7.81, −3.75), *Z* = −5.277, *p* < 0.001; −10.00 (−12.50, −7.50) vs. −5.00 (−7.75, −3.44), *Z* = −4.596, *p* < 0.001]. The level of satisfaction regarding medical services, daily care, and psychological comfort was higher in the experimental group compared to the control group [3 (3, 3.25) vs. 2 (1, 2), *Z* = −5.931, *p* < 0.001; 3 (3, 4) vs. 3 (2, 3), *Z* = −2.286, *p* = 0.022; 2 (1, 3) vs. 1 (0.75, 2), *Z* = −2.081, *p* = 0.037].

**Conclusion:**

In the context of an aging society, telemedicine system can offer improved healthcare to older adults postoperative patients. This includes benefits such as reducing number of visits, saving medical expenditures, enhancing psychological comfort and daily care.

## 1 Introduction

The population aging is accelerating at an unprecedented pace. The World Health Organization (WHO) estimates that by 2050, the proportion of the global population over 60 years will nearly double from 2015, from 12 to 22 percent (1). The process of population aging in China underwent an acceleration phase in the late 1970s and has since consistently grown at an annual rate of approximately 3.2%. Notably, this process that unfolded over more than 45 years in developed nations occurred in just about 27 years in China, and the trend of aging is expected to persist for an extended duration (2). Given the population aging, the growing demand for medical resources has created a certain contradiction between supply and demand with the existing medical resources (3).

Telemedicine, as defined by the WHO, is characterized as “healing from a distance.” To be more precise, it entails the utilization of information and communication technologies to enhance patient outcomes by broadening access to medical care and information (4). A growing awareness is emerging regarding the expansive potential of remote medical care to enrich healthcare services. This approach effectively leverages limited medical resources, enhances the practices of clinical diagnosis, treatment, and disease care, and further bolsters individual health, particularly in developing nations (5). As the largest developing country, remote health services can help address China’s healthcare challenges (6), in particular by alleviating the shortage of healthcare resources and the problem of centralized distribution of healthcare personnel (7). With government support and public recognition, online medical services are rapidly expanding (8).

In the present day, as society advances rapidly, adults’ lives are increasingly consumed by work, especially the middle-aged face undeniable pressures. Home-based rehabilitation, caregiving, and follow-up for older adults postoperative patients are gradually witnessing a reduction in the participation of family members. Older adults individuals who have undergone surgical procedure require ongoing medical services even after being discharged home, including incision dressing change, medication management, dietary guidance, exercise recommendation, health consultation, and various other forms of postoperative care. Compounded by the fact that their children are often not medical professionals, this situation presents challenges to facilitating effective home-based rehabilitation for older adults postoperative patients. In response, our research group has developed a telemedicine system tailored for older adults postoperative patients returning home for rehabilitation. This system aims to provide enhanced convenience and high-quality medical services alongside older adults care.

## 2 Materials and methods

### 2.1 Study design

A double-blinded, randomized controlled trial was conducted from May 2020 to May 2022. Older adults patients (aged ≥65 years) who underwent surgical treatment from Wenzhou Hospital of Integrated Traditional Chinese and Western Medicine were enrolled as participants. A total of 88 participants were subsequently divided into an experimental group and a control group based on disease type, utilizing disease type as a stratification factor through a random number table in a 3:1 ratio. Due to the impact of the COVID-19 pandemic, there had been a reduction in the number of patients seeking surgical treatment, making patient recruitment challenging. Additionally, based on preliminary research indicating a favorable inclination toward remote medical care for patients, the experimental design for this study set a ratio of 3:1 between the experimental and control groups.

The implementers of the two groups were different, including a doctor and a nurse, respectively. The doctor, nurse, and patients in each group only knew the content implemented in their own group, but did not know whether they were in the experimental group or the control group, nor did they know the content implemented in the other group, nor did they know the purpose of this study.

All participants signed a written informed consent. The study protocol was approved by Ethics Committee of Wenzhou Hospital of Integrated Traditional Chinese and Western Medicine. Data and safety monitoring were handled by an independent board.

**Figure 1:**
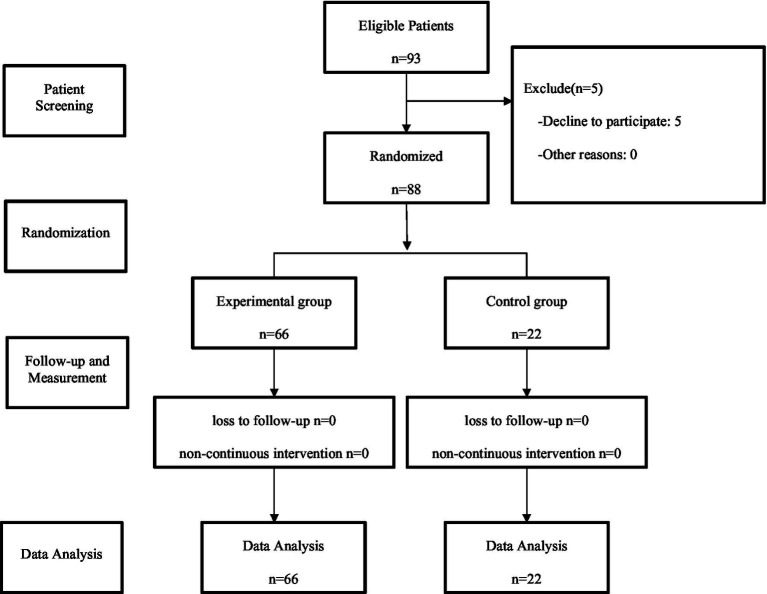
CONSORT flow diagram.

Please refer to Figure 1 for the experimental flowchart.

### 2.2 Inclusion and exclusion criteria

Inclusion Criteria:

1. Stable vital signs during treatment: Patients need to maintain stable vital signs during the treatment.
2. No or slight pain: Patients should not experience significant pain at discharge, or only mild pain.
3. No postoperative complications: No complication arising from surgery was found before discharge.
4. Self-feeding without intravenous nutritional support: Patients should be able to feed themselves without the need for intravenous nutritional support.
5. Normal bowel and bladder function: Postoperative patients’ bowel and bladder functions should be normal.
6. Able to move: Patients should be able to get out of bed and move around.
7. Well-controlled chronic diseases, stable condition: Chronic diseases of patients should have been well-controlled, and their overall health should be stable.
8. Agree to be discharged: Patients should agree to be discharged.
9. Possession of a smartphone or relevant device for video call: Patients need to have a smartphone or a relevant device capable of video call.
10. At least one accompanying family member, agreement on remote medical services by the patient and family: Patients need to have at least one accompanying family member, and both the patient and their family must agree to receive remote medical services.

Exclusion Criteria:

1. Presence of surgical contraindications: Patients with contraindications for surgery will be excluded.
2. History of mental illness for the patient or primary caregiver: Patients or their primary caregivers with a history of mental illness will be excluded from the study.

### 2.3 Intervention

The experimental group integrated “medical services” and “older adults care” through the utilization of remote diagnosis and treatment application and communication software such as WeChat application.

#### 2.3.1 Pre-discharge training

Upon participants meeting the discharge criteria and expressing consent, healthcare professionals conducted basic training for their caregivers. If the primary caregiver encountered challenges in learning, another eligible family member was assigned to receive appropriate training. The training encompassed the following areas: (A) Follow-up plan. (B) Utilization of telemedicine application and communication software. (C) Guidance for changing incision dressing. (D) Medication, diet, and exercise guidance. (E) Matters needing attention. (F) Home blood pressure and/or blood sugar measurement.

#### 2.3.2 Medical services

Healthcare professionals conducted remote consultations with patients and their families using communication software on the 3rd, 7th, and 14th day after discharge. The services encompass: (A) Collecting information about older adults patients’ symptoms, as well as some basic physical signs, including mental status, consciousness, complexion, abdominal appearance, and wound healing. (B) Providing real-time guidance to caregivers during incision dressing changes. (C) Emphasizing key aspects of postoperative rehabilitation. (D) Managing chronic conditions by reviewing recent measurements such as temperature, respiration, heart rate, blood pressure, and blood sugar. Offering guidance on adjusting medication, diet, and exercise plans through remote communication software. Highlighting the significance of chronic disease management and follow-up protocol. (E) Establishing a direct communication channel for promptly reporting of any discomfort or abnormalities in older adults postoperative patients. Simplifying hospital visits through a streamlined process, including prearranged appointments. (F) Facilitating online prescription issuance through the remote diagnosis and treatment application and communication software, followed by offline delivery of prescribed medications via Express.

#### 2.3.3 Daily care

Personalized dietary and exercise plans were crafted to align with distinct health conditions and surgical interventions. The postoperative regimen should delineate dietary specifics, encompassing the avoidance of alcoholic beverages and spicy foods. Additionally, during the rehabilitation process, patients were reminded of relevant precautions through follow-up. Individuals who had undergone gallbladder removal were advised to moderate their intake of fatty foods to mitigate the risk of diarrhea. For patients recuperating from appendectomy or gallbladder surgery, appropriate activity was recommended to expedite the recovery of gastrointestinal functionality. Similarly, individuals undergoing surgery for great saphenous vein varicose and inguinal hernia should refrain a prolonged walk, aside from their routine activities, to avert leg swelling and hernia patch displacement. The relevant content was also presented and explained to patients and their families through text, pictures, or videos. This approach was geared toward enhancing comprehension, reinforcing information retention, and fostering adherence.

#### 2.3.4 Psychological comfort

Throughout the follow-up process, the older adults postoperative patients received continuous encouragement and reassurance to bolster their confidence in postoperative recovery. Timely psychological counseling was provided for those displaying noticeable anxiety or depression, with psychological assistance readily available when required. Additionally, family members were encouraged to engage actively, provide attentive care, and offer patient guidance.

The control group underwent “medical services” and “older adults care” interventions according to the traditional protocol. In terms of medical services, healthcare professionals conducted telephone follow-ups with the older adults postoperative patients on the 3rd, 7th, and 14th day after discharge. The primary focus was to assess postoperative recovery and management of chronic conditions among the older adults postoperative patients. Whenever necessary, guidance was provided, and recommendations were made for hospital or local medical care. Incision dressing changes could be performed at our hospital or a local healthcare facility. Regarding “older adults care” aspects, healthcare professionals only provided some advices.

### 2.4 Study outcomes

Both groups underwent evaluation on the following indices. The experimental group was assessed using remote communication software, while the control group was assessed using telephone. To ensure impartiality, two nurses who were unaware of the study’s methods and objectives were assigned by the research group to conduct the evaluation.

#### 2.4.1 Number of medical visits and medical expenditures

One month after discharge, the number of medical visits and associated medical expenditures were analyzed. The count encompassed the number of visits each older adults person made to medical facilities, along with the computation of the medical expenditures incurred during these visits.

#### 2.4.2 Postoperative recovery

One month after discharge, the occurrence of incision infection, incision bleeding, incision dehiscence, readmission, and reoperation was quantified in both groups. Additionally, physical status was assessed using the Karnofsky Performance Scale (KPS) (9) at discharge and one month after discharge, and body weight change was calculated by subtracting the weight at discharge from the weight one month after discharge.

#### 2.4.3 Anxiety and depression

At discharge and one month after discharge, the Zung Self-Rating Anxiety Scale (SAS) (10) and Self-Rating Depression Scale (SDS) (11) were employed to assess the level of anxiety and depression of each older adults postoperative patient.

#### 2.4.4 Satisfaction

One month after discharge, the satisfaction of the older adults postoperative patients was evaluated. The evaluation encompassed three facets: medical services, daily care, and psychological comfort. Each facet was evaluated using the Likert 5-point grading method (12, 13), encompassing responses from “very dissatisfied,” “dissatisfied,” “neutral,” “satisfied,” to “very satisfied,” each assigned a score ranging from 0 to 4 points.

### 2.5 Statistical analysis

All study data were analyzed using SPSS 18.0 software. The *t*-test was employed to compare means in normally distributed data between the two groups. The Pearson Chi-square test, Fisher Chi-square test, or Fisher–Freeman–Halton Chi-square test was used to assess differences in count data between the two groups. For rank data and non-normally distributed data, the Mann–Whitney U rank-sum test was utilized for comparison. The two-sided tests were used in this study and a significance level of *p* < 0.05 was considered statistically significant.

## 3 Results

### 3.1 Characteristics of the participants

The experimental group comprised 66 participants, of which 36 (54.5%) were males and 30 (45.5%) were females, with an average age of 71.29 ± 5.20 years. In terms of educational background, there were 10 (15.2%) participants with high school education, 21 (31.8%) with middle school education, and 35 (53.0%) with primary school education or lower. Among these participants, 27 (40.9%) underwent inguinal hernia surgery, 15 (22.7%) had varicose great saphenous vein surgery, 15 (22.7%) had appendix surgery, and 9 (13.6%) underwent gallbladder surgery. In terms of past history, it included 15 (22.7%) cases of hypertension, 10 (15.2%) cases of diabetes mellitus and 8 (12.1%) cases of other chronic diseases. In the control group, there were 22 participants, including 13 (59.1%) males and 9 (40.9%) females, with an average age of 72.09 ± 5.03 years. In terms of educational background, there were 3 (13.6%) participants with high school education, 6 (27.3%) with middle school education, and 13 (59.1%) with primary school education and lower. Among them, 9 (40.9%) underwent inguinal hernia surgery, 5 (22.7%) had varicose great saphenous vein surgery, 5 (22.7%) had appendix surgery, and 3 (13.6%) underwent gallbladder surgery. Moreover, the control group consisted of 7 (31.8%) patients with hypertension, 3 (13.6%) with diabetes, and 3 (13.6%) with other chronic illnesses. Notably, there was no significant difference in basic demographic characteristics between the two groups (*p* > 0.05). For detailed information, please refer to Table 1.

**Table 1:** Basic information of the two groups.

	Experimental group (*n* = 66)	Control group (*n* = 22)	*χ*^2^/*t*/*Z*	*p*-value	95% CI lower limit/upper limit
**Gender** ^**#**^					
Male	36 (54.5%)	13 (59.1%)	0.138	0.710	
Female	30 (45.5%)	9 (40.9%)			
Age (years)*	71.29 ± 5.20	72.09 ± 5.03	−0.632	0.529	−3.328/1.722
**Education level** ^**†**^					
Primary school	35 (53.0%)	13 (59.1%)	−0.456	0.648	
Junior high school	21 (31.8%)	6 (27.3%)			
High school and above	10 (15.2%)	3 (13.6%)			
**Type of operation** ^**¤**^					
Inguinal hernia	27 (40.9%)	9 (40.9%)	0.347	1.000	
Great saphenous vein	15 (22.7%)	5 (22.7%)			
Appendix	15 (22.7%)	5 (22.7%)			
Gall bladder	9 (13.6%)	3 (13.6%)			
**Chronic disease** ^**¤**^					
High blood pressure	15 (22.7%)	7 (31.8%)	1.067	0.801	
Diabetes	10 (15.2%)	3 (13.6%)			
Else	8 (12.1%)	3 (13.6%)			
None	33 (50.0%)	9 (40.9%)			

Data are presented as numbers with percentages (%) or mean ± standard deviation.

^#^Categorical variables were compared using Person chi-square test.

^¤^Categorical variables were compared using Fisher–Freeman–Halton chi-square test.

^*^Normally distributed data were compared using *t*-test.

^†^Rank data were compared using the Mann–Whitney U rank sum test.

### 3.2 Medical visits and medical expenditures

The number of visits of the experimental group was 1 (0, 1), whereas that of the control group was 1 (1, 2). The difference between the two groups was statistically significant (*Z* = −3.977, *p* < 0.001).

The medical expenditures of the experimental group amounted to 25.25 (0.00, 277.40) yuan, whereas that of the control group amounted to 174.65 (49.63, 446.10) yuan. The difference between the two groups was statistically significant (*Z* = −2.150, *p* = 0.032) (see Table 2 for details).

**Table 2:** Number of visits and medical expenditures of the two groups.

	Experimental group (*n* = 66)	Control group (*n* = 22)	/*Z*	*p*-value
Number of visits	1 (0, 1)	1 (1, 2)	−3.977	<0.001
Medical expenditures (yuan)	25.25 (0.00, 277.40)	174.65 (49.63, 446.10)	−2.150	0.032

Data are presented as median and quartile (P25, P75).

Non-normally distributed data were compared using the Mann–Whitney U rank sum test.

### 3.3 Postoperative recovery

Two cases of incision infection occurred in each of the two groups, and there was no statistically significant difference between the two groups (Fisher χ^2^, *p* = 0.259). Notably, no instance of incision bleeding, incision dehiscence, readmission, or reoperation was reported in either of the two groups. There was no significant difference in physical status between the two groups at discharge and after discharge (66.06 ± 8.92 vs. 65.45 ± 7.39, *t* = 0.287, *p* = 0.775; 73.33 ± 9.97 vs. 70.91 ± 7.50, *t* = 1.202, *p* = 0.235). And there was no significant difference in the change of physical status between the two groups after discharge [10.00 (0.00, 10.00) vs. 5.00 (0.00, 10.00), *Z* = −1.077, *p* = 0.281]. Additionally, there was no significant difference in body weight change between the two groups after discharge [1.05 (0.38, 1.60) Kg vs. 0.80 (0.50, 1.43) Kg, *Z* = −0.265, *p* = 0.791] (see Table 3 for details).

**Table 3:** Physical status and body weight change of the two groups.

	Experimental group (*n* = 66)	Control group (*n* = 22)	*t/Z*	*p*-value	95% CI lower limit/upper limit
Physical status at-discharge^*^	66.06 ± 8.92	65.45 ± 7.39	0.287	0.775	−3.590/4.801
Physical status post-discharge^*^	73.33 ± 9.97	70.91 ± 7.50	1.202	0.235	−1.630/6.479
Change of physical status^#^	10.00 (0.00, 10.00)	5.00 (0.00, 10.00)	−1.077	0.281	
Body weight change (Kg)^#^	1.05 (0.38, 1.60)	0.80 (0.50, 1.43)	−0.265	0.791	–

Data are presented as mean ± standard deviation or median and quartile (P25, P75).

The change of physical status is calculated by subtracting the physical status score at discharge from the physical status score after discharge.

^*^Normally distributed data were compared using *t*-test.

^#^Non-normally distributed data were compared using the Mann–Whitney U rank sum test.

### 3.4 Anxiety and depression

There was no significant difference in the levels of anxiety and depression between the two groups at discharge (45.64 ± 8.10 vs. 44.60 ± 8.24, *t* = 0.520, *p* = 0.604; 48.33 ± 8.46 vs. 47.50 ± 6.85, *t* = 0.418, *p* = 0.677). But the levels of anxiety and depression in the experimental group were lower than those in the control group after discharge (34.92 ± 7.38 vs. 39.03 ± 8.42, *t* = −2.183, *p* = 0.032; 37.86 ± 7.29 vs. 41.93 ± 7.13, *t* = −2.281, *p* = 0.025); The change of Anxiety level and Depression level of the experimental group were more than those of the control group [−10.00 (−11.25, −8.75) vs. −5.00(−7.81, −3.75), *Z* = −5.277, *p* < 0.001; −10.00 (−12.50, −7.50)vs. −5.00(−7.75, −3.44), *Z* = −4.596, *p* < 0.001] (see Table 4 for details).

**Table 4:** Anxiety and depression of the two groups.

	Experimental group (*n* = 66)	Control group (*n* = 22)	*t/Z*	*p*-value	95% CI lower limit/upper limit
Anxiety at discharge^*^	45.64 ± 8.10	44.60 ± 8.24	0.520	0.604	−2.941/5.024
Anxiety after discharge^*^	34.92 ± 7.38	39.03 ± 8.42	−2.183	0.032	−7.853/−0.367
Change of anxiety^#^	−10.00 (−11.25, −8.75)	−5.00 (−7.81, −3.75)	−5.277	<0.001	
Depression at discharge^*^	48.33 ± 8.46	47.50 ± 6.85	0.418	0.677	−3.128/4.795
Depression after discharge^*^	37.86 ± 7.29	41.93 ± 7.13	−2.281	0.025	−7.620/−0.524
Change of depression^#^	−10.00 (−12.50, −7.50)	−5.00 (−7.75, −3.44)	−4.596	<0.001	

Data are presented as mean ± standard deviation or median and quartile (P25, P75).

The Change of anxiety/depression is calculated by subtracting the anxiety/ depression score at discharge from the anxiety/depression score after discharge.

^*^Normally distributed data were compared using *t*-test.

^#^Non-normally distributed data were compared using the Mann–Whitney U rank sum test.

### 3.5 Patient satisfaction

The level of satisfaction regarding medical services, daily care, and psychological comfort was higher in the experimental group compared to the control group [3 (3, 3.25) vs. 2 (1,2), *Z* = −5.931, *p* < 0.001; 3 (3, 4) vs. 3 (2, 3), *Z* = −2.286, *p* = 0.022; 2 (1, 3) vs. 1 (0.75, 2), *Z* = −2.081, *p* = 0.037] (see Table 5 for details).

**Table 5:** Satisfaction level of the two groups.

	Experimental group (*n* = 66)	Control group (*n* = 22)	*Z*	*p*-value
Medical services	3 (3, 3.25)	2 (1, 2)	−5.931	<0.001
Daily care	3 (3, 4)	3 (2, 3)	−2.286	0.022
Psychological comfort	2 (1, 3)	1 (0.75, 2)	−2.081	0.037

Data are presented as median and quartile (P25, P75).

Non-normally distributed data were compared using the Mann–Whitney U rank sum test.

## 4 Discussion

### 4.1 The telemedicine system can better meet the medical requirements of older adults postoperative patients

The study revealed that older adults postoperative patients in the experimental group made fewer hospital visits compared to those in the control group, resulting in lower medical costs. This indicates that the remote medical care model is more effective in addressing the medical requirements of older adults postoperative patients.

Based on a systematic review, an individual’s social, cultural, and digital technological competence is a crucial factor in determining the benefits derived from telemedical care (14). Consequently, this study diligently endeavored to impart suitable training to patients and their families enrolled in the telemedicine system. Caregivers were ultimately empowered and assist older adults in changing incision dressings independently or under the guidance of a telemedicine specialist.

When an older adults individual was feeling unwell, a medical professional can conduct a remote assessment. Certain symptoms could be addressed through temporary observation, modifications to diet/exercise, or suitable medication, effectively eliminating the necessity for a hospital visit. This approach served to alleviate patient anxiety and also helps in minimizing unnecessary visits. As for the no difference in recovery between the two groups, this might be related to the insufficient sample size.

About 65% of the older adults suffered from various chronic diseases (15), such as hypertension (16) and diabetes (17, 18). Consequently, while the telemedicine system devised in this study primarily targeted the enhancement of rehabilitation among the older adults postoperative patients, it also encompassed an indispensable facet of chronic disease management. The study revealed instances where blood pressure and blood sugar levels of certain chronic patients in the control group were inadequately managed after discharge. In contrast, the experimental group benefited from the guidance provided by medical professionals, resulting in more favorable outcomes. A study demonstrated that regular Blood Pressure Tracking (BPT), compared to conventional treatment, could significantly lower blood pressure, particularly for high-risk hypertensive patients (19, 20). Coincidentally, remote medical care was also advantageous for controlling blood sugar levels in diabetic patients (21). In the telemedicine system established in this study, well-trained caregivers played an important role in routinely monitoring the blood pressure and blood sugar of the older adults.

Additionally, this service model provided convenience for medical staff to adjust treatment and care plans in a timely manner based on changes in patients’ conditions, such as providing remote guidance or supervision in areas such as diet, exercise, and medication treatment. The study by Barbosa et al. (22) also indicated that satisfactory treatment outcomes could be achieved through network-enabled remote management of chronic diseases. Generally, by effectively utilizing smart devices and the WeChat platform, it was possible to further enhance the accessibility of medical services and the quality of healthcare for surgical patients and their families. This aligned with the findings of previous researches (23–25).

### 4.2 The telemedicine system can better meet the psychological requirements of older adults postoperative patients

The occurrence of COVID-19 pandemic could lead to anxiety, depression, and other adverse emotions among the older adults. Particularly, older adults who had undergone surgical treatment were more susceptible to experiencing anxiety and depression (26). This psychological vulnerability arised from concerns not only about postoperative rehabilitation but also the management of chronic diseases. In instances where a patient experienced excessive worry about their condition, medical personnel could address the concerns of the older adults through an objective assessment of the situation. In our actual research, we discovered that the older adults postoperative patients, to varying degrees, tended to experience concerns about the successfulness of their recovery following discharge. These concerns often revolved around issues such as medication adjustment plan, time for follow-up appointment, dietary restriction, optimal level of physical activity, and permissibility of bathing. Even after receiving answers to these recurring queries, they might still raise the same question again. Such a scenario was likely indicative of anxiety. An experiment involving remote medical care for older adults patients indicated that among the participants receiving remote medical care, 36% of patients required essential technical assistance, while 58% of patients needed additional psychological support due to a lack of confidence (27).

Within this system, caregivers of the older adults underwent fundamental training. This equipped them with an understanding of the older adult’s condition and post-discharge care requirements, enabling them to offer explanations to the older adults, replacing the need for repeated involvement of medical staff. Furthermore, video calls between healthcare professionals and the older adults within this system might be more effective in enhancing patients’ psychological well-being than telephone calls in the control group. For instance, medical staff could distinctly perceive the expressions of the older adults and foster a stronger sense of trust between them, consequently facilitating medical staff to provide more effective psychological counseling for the older adults. Similarly, Lim et al. (28) also discovered in their research on remote chronic disease management that this approach could effectively alleviate anxiety and depression among older adults patients. This finding corresponded with the conclusions drawn from our study. This indicated that the widespread adoption of mobile technology could alleviate patients’ psychological health needs (29).

### 4.3 The telemedicine system can improve the satisfaction of older adults postoperative patients

The study revealed that the older adults postoperative patients in the experimental group exhibited higher levels of satisfaction with medical services, daily care, and psychological comfort compared to those in the control group. This observation suggested that the telemedicine system had a more favorable impact in these particular areas.

#### 4.3.1 Medical services

Collaborative efforts between doctors and nurses within the telemedical system resulted in improved the effectiveness of postoperative rehabilitation guidance and enhanced chronic disease management for the older adults. This, in turn, leaded to heightened satisfaction with medical services among the older adults. A retrospective survey indicated that telemedicine was found satisfactory on various outcome and the most common advantages were time saved (30).

#### 4.3.2 Daily care

Within the context of the telemedicine system, family members engaged in remote follow-up, enabling them to gain a deeper understanding of the older adults postoperative patients’ physical and mental well-being, and subsequently provided meticulous care. Additionally, the remote follow-up by medical staff also served as a catalyst for motivating family members, thereby potentially leading to more standardized and refined daily care.

#### 4.3.3 Psychological comfort

The telemedicine system harmonized the involvement of medical staff and family members in the postoperative management of the older adults. This collaborative approach helped to gain better insight into the psychological states of the older adults. When necessary, prompt psychological counseling could be offered, resulting in increased psychological comfort for the older adults. Furthermore, the combined effect of enhanced daily care and improved medical services can contribute to alleviating anxiety and depression among older adults individuals. Existing research indicated that mobile technology-based applications not only facilitated familial connectivity but also established a connection between the older adults and healthcare resources, fostering enhancements in both physical and mental well-being (21).

In this study, as all patients were older adults, the scoring sheet was designed to request simple ratings directly from patients only in the aspects of medical services, daily care, and psychological comfort. Because each aspect was evaluated using a single item, we did not measure the reliability and validity of the questionnaire. To ensure the stability of the measurement, the measurements of both groups were carried out by the same personnel, and the same understandable text expression and language explanation of item was used during the measurement. In future research, adding rating items may enhance scientific validity of data.

But this study only discusses patients who can participate in telemedicine system, and for some older adults individuals, the digital divide may pose a limitation. In cases where patients are unable to access the telemedicine system due to various reasons, it is crucial to establish support measures for telemedicine system management. Firstly, design a user-friendly interface that meets the unique needs and preferences of older adults users. Additionally, provide clear and concise instructions on how to access telemedicine platforms and resources. Implementing user training courses or tutorials may further enhance digital literacy among the older adults population. Moreover, establish a dedicated support system, such as a hotline or online assistance, to address any technological challenges or concerns. By taking these measures, healthcare providers can optimize the use of telemedicine tools, improving the overall experience and outcomes for older adults postoperative patients. Proficient individuals, utilizing smart devices, can access fundamental daily care and rehabilitation procedures through the hospital’s social platforms. This not only alleviates the workload of doctors but also enhances the quality of medical services (31).

## 5 Conclusion

In summary, the integrated telemedicine model developed in this study holds in line with the contemporary landscape of increasing aging population. This model amalgamates medical services and older adults care, facilitating older adults postoperative patients to curtail needless medical visits while experiencing top-notch healthcare provisions within community settings. This approach fosters the physical and psychological wellness of older adults postoperative patients. Consequently, advocating for its broader dissemination and adoption is justified. To foster the advancement of telemedicine, governmental agencies must enhance regulation and integration, while the healthcare system should offer support in personnel and software systems. Importantly, it is necessary to amplify public awareness and acceptance of remote medical care. The comprehensive development of telemedicine demands collaborative efforts from diverse sectors.

## Data availability statement

The raw data supporting the conclusions of this article will be made available by the authors, without undue reservation.

## Ethics statement

The studies involving humans were approved by Wenzhou Hospital of Integrated Traditional Chinese and Western Medicine Ethics Committee. The studies were conducted in accordance with the local legislation and institutional requirements. The participants provided their written informed consent to participate in this study.

## Author contributions

Q-PW: Conceptualization, Data curation, Investigation, Writing – original draft. W-YC: Conceptualization, Data curation, Investigation, Writing – original draft. M-MH: Data curation, Formal analysis, Writing – original draft. Y-XH: Data curation, Formal analysis, Writing – original draft. S-SL: Formal analysis, Writing – review & editing. Y-CG: Formal analysis, Writing – review & editing.
